# Having Your Cake and Eating It Too: Combining SBRT and Multi-agent Chemotherapy in Locally Advanced Pancreatic Cancer

**DOI:** 10.7759/cureus.686

**Published:** 2016-07-13

**Authors:** Amar Kishan, Percy Lee

**Affiliations:** 1 Department of Radiation Oncology, University of California, Los Angeles

**Keywords:** sbrt, pancreatic cancer, lap-07

## Abstract

We read with great interest the results of the LAP07 study comparing capecitabine-based chemoradiation with gemcitabine-based therapy for non-progressive locally advanced pancreatic cancer (LAPC), following four months of gemcitabine-based therapy. The results, consistent with previous data, showed that standard chemoradiation improves local control (LC) but not overall survival. In this brief editorial, we emphasize that LC may still be very important in LAPC, as up to 30% of patients with LAPC may die from locally progressive disease. This is particularly likely to be true as systemic therapies continue to improve in efficacy. We very briefly review the data in support of stereotactic body radiotherapy (SBRT) for LAPC, which has been shown to offer excellent LC with minimal late grade ≥ 2 toxicity rate in a recent multi-institutional phase II study. We underscore that  a short course of SBRT offers an expeditious alternative to a long course of chemoradiation, allowing the use of fully-intensive systemic therapy.

## Editorial

We read with great interest the results of the LAP07 study comparing capecitabine-based chemoradiation with gemcitabine-based therapy for non-progressive locally advanced pancreatic cancer (LAPC), following four months of gemcitabine-based therapy [1]. The results, consistent with previous data, showed that standard chemoradiation improves local control (LC), but not overall survival.

One must be cautious, however, to ‘not throw the baby out with the bathwater’—we submit that LC is still important in LAPC. Up to 30% of patients with LAPC die from locally progressive disease [2]. For patients with localized disease and those whose metastatic burden is controlled by effective systemic therapy, LC may be particularly important. The authors astutely discussed that advanced radiotherapy techniques may afford better LC. This is particularly relevant, as LAPC can be heterogeneous not only with regards to a proclivity for metastatic vs. local spread but also with regards to radiosensitivity. Intriguingly, stereotactic body radiotherapy (SBRT), which allows delivery of ablative radiotherapy in ≤ 5 sessions, has demonstrated excellent LC for LAPC [3-4]. The Stanford experience utilizing a single fraction of 25 Gy (with or without conventionally fractionated radiation, primarily in patients treated receiving gemcitabine-based chemotherapy) found an overall LC rate of 84% at 12 months [3]. Enthusiasm for this approach was tempered by the 25% 12-month actuarial rate of late grade ≥ 2 gastrointestinal toxicity. Subsequent multi-fraction SBRT schedules have yielded improved toxicity profiles. For example, a recent phase II multi-institutional study evaluating a dose of 33 Gy in five fractions after up to three weeks of gemcitabine-based therapy reported a 12-month LC rate of 78%, with an attendant late grade ≥ 2 toxicity rate of only 11% [4]. Continued technical advancements in radiotherapy delivery may further reduce toxicity by sparing normal tissues, such as the adjacent duodenum.

Because SBRT is delivered in ≤ 5 fractions, it allows patients to quickly resume systemic therapy, eliminating the delay caused by conventionally fractionated chemoradiation [5]. Patients with non-progressive disease on optimal systemic therapy regimens may receive SBRT and then resume systemic therapy in short order. This strategy is being tested in a multi-institutional prospective phase III study (https://clinicaltrials.gov/ct2/show/NCT01926197) in which patients with non-progressive disease following up to four cycles of FOLFIRINOX are randomized to receive SBRT (40 Gy in 5 fractions) or further FOLFIRINOX. We encourage the oncology community to support this effort to determine if SBRT, incorporated with multi-agent systemic therapy, may improve overall survival. Hopefully, continued investigation will determine the optimal sequencing and combination of systemic and local therapy for patients with LAPC. 

We have no conflicts of interest to disclose.
